# A Layman's Opinion

**Published:** 1921-12

**Authors:** 


### CORRESPONDENCE.

[Correspondence on all subjects is invited, but we cannot in any way be responsible for the opinions expressed by our correspondents, who must give their name and address as a guarantee of good faith, but not necessarily for publication. Correspondents are reminded that brevity of style and conciseness of statement greatly facilitate early insertion.]

### A Layman's Opinion.

*To the Editor of* THE HOSPITAL AND HEALTH REVIEW.

SIR,—I was given a copy of your paper by the secretary to our local hospital, and you may be pleased to hear that I found it quite interesting. . . Of course the article on the Black Death was all right in its way, but is it any good to us to know about things that happened hundreds of years ago? . . . We don't get Black Deaths now. And why don't we get them? Because we are clean is my answer to that. . . .—Yours, etc.,

"MIDLAND."

[We regret that we have been obliged to condense this correspondent's letter, which otherwise would have occupied three columns. In spite of his assertion, we *do* get Black Deaths now—there was pneumonic plague in Manchuria in 1910, and there seems to be an increase of plague in the East at the present time.—EDITOR, H. & H.R.]

